# In-Vitro Effects of Perfluorooctanoic Acid on Human Sperm Function: What Are the Clinical Consequences?

**DOI:** 10.3390/jcm13082201

**Published:** 2024-04-11

**Authors:** Angela Alamo, Sandro La Vignera, Laura M. Mogioì, Andrea Crafa, Federica Barbagallo, Rossella Cannarella, Antonio Aversa, Aldo E. Calogero, Rosita A. Condorelli

**Affiliations:** 1 Department of Clinical and Experimental Medicine, University of Catania, Via S. Sofia 78, 95123 Catania, Italy; angela.alamo1986@gmail.com (A.A.); lauramongioi@hotmail.it (L.M.M.); crafa.andrea@outlook.it (A.C.); federica.barbagallo11@gmail.com (F.B.); rossella.cannarella@phd.unict.it (R.C.); acaloger@unict.it (A.E.C.); rosita.condorelli@unict.it (R.A.C.); 2 Department of Experimental and Clinical Medicine, University Magna Graecia of Catanzaro, 88100 Catanzaro, Italy; aversa@unicz.it

**Keywords:** PFOA, sperm motility, bio-functional sperm parameters, oxidative stress, DNA integrity

## Abstract

**Background**: Lifestyle and environmental pollution harm male fertility. Perfluoroalkyl substances (PFAS) are bio-accumulates in the environment as well as in several human tissues, and one of the most common PFAS is perfluorooctanoic acid (PFOA). Therefore, this study aimed to evaluate the in vitro effects of PFOA with hydrophobic and waterproofing properties on motility and bio-functional sperm parameters. **Methods**: To accomplish this, 50 healthy men with normozoospermia and not exposed to high doses of PFAS were enrolled. Their spermatozoa were incubated for 3 h with increasing concentrations of PFOA (0, 0.01, 0.1, and 1 mM) to evaluate its effects. In particular, we evaluated the effects of PFOA on total and progressive sperm motility and, by flow cytometry, on the following bio-functional sperm parameters: degree of chromatin compactness, viability, early and late apoptosis, mitochondrial membrane potential, the degree of lipoperoxidation, and concentrations of mitochondrial superoxide anion. **Results**: The results showed that PFOA decreased both total and progressive sperm motility, impaired chromatin compactness, and increased sperm lipid peroxidation and mitochondrial superoxide anion levels. **Conclusions**: This study showed that PFOA alters several sperm parameters and thus it may play a negative role in male fertility.

## 1. Introduction

The effects of environmental pollution on sperm quality have been investigated but the data remain inconclusive [1,2]. Environmental factors that potentially affect male fertility include cigarette smoking, exposure to pesticides and toxins, and radiofrequency electromagnetic radiation [3,4,5,6,7]. In previous studies, we have shown the effects of toxic compounds such as nicotine or benzo-a-pyrene on sperm quality [8,9]. Few studies have been published on perfluoroalkyl substances and their effects on human and reproductive health are not well known.

Perfluoroalkyl substances (PFAS) are ubiquitous substances characterized by fluorinated hydrocarbon chains that confer chemical and thermal stability. These properties allow their use in industry and consumer products, including oil and water repellents, coatings for cookware, carpets, and textiles [10]. The presence of long chains makes these compounds non-biodegradable. Therefore, they bio-accumulate in the environment [11] as well as in several human tissues, such as plasma, brain, placenta, testis, and semen even in the presence of specific barriers [12,13,14]. One of the most common PFAS is perfluorooctanoic acid (PFOA) that is used to produce non-stick pans [15].

PFOA has a long half-life of approximately 3.5 years [16] and it has been classified as “possibly carcinogenic to humans” (Group 2B) by the International Agency for Research on Cancer, World Health Organization [17]. In areas with elevated contamination levels of PFAS, the amount of PFOA measured in drinking water is >1000 times higher than the values considered normal (from 0.5 to 8 ng/L). Moreover, it is found in the blood and semen fluid at levels that are >5-fold higher than those of men living in areas at a lower level of contamination [18]. Despite the evidence of the negative role of PFAS on human reproductive health and their role as endocrine disruptors [18,19,20,21], few studies have evaluated the effects of PFAS on sperm parameters and the results are somewhat conflicting [22,23,24]. 

Likewise, studies on the effects of PFAS on DNA fragmentation have not been conclusive. 

Therefore, the aim of our study was not only to evaluate the in vitro effects of PFOA on human sperm parameters but also to evaluate the bio-functional parameters (DNA fragmentation, degree of chromatin compactness, viability, early and late apoptosis, mitochondrial membrane potential, lipid peroxidation, and mitochondrial superoxide) after incubation. To accomplish this, spermatozoa collected from healthy donors were incubated with increasing concentrations of PFOA to evaluate its effect on sperm motility and bio-functional sperm parameters.

## 2. Materials and Methods

### 2.1. Patient Selection

The study was conducted on 50 healthy men (mean age 34.6 ± 3.2 years) attending the Division of Andrology and Endocrinology, University of Catania, for sperm analysis. 

A total of 77 men were surveyed but only 50 of them met the requirements for enlistment. We recruited men with normal sperm parameters, who answered a questionnaire that included information on previous or current diseases, including any known history of fertility potential and relevant lifestyle factors such as smoking and drinking habits [21]. In addition to excluding the marked exposure to PFAS, patients provided information on their geographical residence, occupation, and diet. The participants underwent an accurate medical examination, measurements of the anthropometric parameters, testicular ultrasound scan, and semen analysis. Only men not highly exposed to PFOA were selected for this study. Patients with male accessory glands infection, systemic diseases, micro-orchid (testicular volume < 12 mL), cryptorchidism, varicocele, and/or who received hormonal treatment in the last 12 months were excluded. 

The investigation was performed according to the principles of the Declaration of Helsinki. The protocol was approved by the Institutional Review Board. Informed written consent was obtained from each man.

### 2.2. Sperm Preparation

Sperm analysis was conducted according to the WHO criteria [25]. 

### 2.3. Chemicals

PFOA was purchased from Sigma-Aldrich s.r.l. (Milan, Italy) and dissolved in DMSO to prepare the mother solution.

### 2.4. Experimental Design

Spermatozoa (aliquots of 1 × 10^6^) were incubated with increasing concentrations of PFOA, at 37 °C, for 3 h. The concentrations of 0.01, 0.1, and 1 mM were chosen according to previously published studies [26,27]. At the end of the incubation, spermatozoa were analyzed to evaluate the effects of PFOA on sperm motility and bio-functional parameters. In particular, we examined total and sperm progressive motility, according to the WHO 2010 criteria, and, by flow cytometry, the following bio-functional parameters: the degree of chromatin compactness, viability, early apoptosis by externalization of phosphatidylserine (PS), late apoptosis, mitochondrial membrane potential (MMP), lipid peroxidation, and mitochondrial superoxide.

### 2.5. Flow Cytometry Analysis

Flow cytometry analysis was performed using flow cytometer CytoFLEX (Beckman Coulter Life Science, Milan, Italy) equipped with two argon lasers and six total fluorescence channels (four 488 nm and two 638 nm). Then, 100,000 events were measured for each sample and analyzed by the software CytExpert 1.2. All methods were performed in accordance with the most relevant guidelines [28].

### 2.6. Assessment of the Degree of Chromatin Compactness

The evaluation of chromatin compactness was performed after the permeabilization of the cell membrane to get the fluorophore to the nucleus. An aliquot of 1 × 10^6^ spermatozoa was incubated with LPR DNA-Prep Reagent (Beckman Coulter, Milan, Italy), in the dark, at room temperature for 10 min and then further incubated with Stain DNA-Prep Reagent containing 50 µg/mL of propidium iodide (PI) (<0.5%), RNase A (4 Kunitz/mL), <0.1% NaN_3_, saline, and stabilizers (Beckman Coulter, Chicago, IL, USA) in the dark at room temperature. Flow cytometry analysis was performed after 30 min.

### 2.7. Evaluation of Sperm Apoptosis/Vitality

The exposure of PS on the outer cell surface is an early signal of apoptosis. The assessment of PS externalization was performed using FITC-labeled annexin V, a protein that binds selectively to the PS. The simultaneous cell staining with PI allows us to distinguish alive spermatozoa (with intact cytoplasmic membrane) and apoptotic or necrotic spermatozoa. An aliquot containing 0.5 × 10^6^/mL was suspended in 0.5 mL buffer containing 10 µL of annexin V-FITC and 20 µL of PI (Annexin V-FITC Apoptosis, Beckman Coulter, Milan, Italy) and incubated for 10 min in the dark. After incubation, the sample was analyzed immediately. The different patterns of staining allowed us to identify the three different cell populations: viable cells (FITC negative and PI negative); cells in early apoptosis with cytoplasmic membrane still intact (FITC positive and PI negative); and cells in late apoptosis (FITC positive and PI-positive).

### 2.8. Evaluation of the Mitochondrial Membrane Potential

MMP was evaluated by a lipophilic probe 5,5′,6,6′-tetrachloro-1,1′,3,3′-tetraethyl-benzimidazolylcarbocyanine iodide (JC-1, DBA s.r.l., Milan, Italy) able to penetrate into mitochondria. Briefly, an aliquot containing 1 × 10^6^/mL of spermatozoa was incubated with JC-1 in the dark, for 10 min, at 37 °C. At the end of the incubation period, the cells were washed in PBS and analyzed. Therefore, the fluorescence changed reversibly from green to orange when the mitochondrial membrane became more polarized. In viable cells with normal membrane potential, JC-1 was in the mitochondrial membrane in the form of aggregates emitting in an orange fluorescence, while in the cells with low membrane potential it remained in the cytoplasm in a monomeric form, giving a green fluorescence.

### 2.9. Evaluation of Sperm DNA Fragmentation

DNA fragmentation was evaluated by the TUNEL assay (Apoptosis Mebstain, DBA s.r.l., Milan, Italy). This kit exploits the action of the Terminal deoxynucleotidyl Transferase (TdT), an enzyme that polymerizes, at the level of DNA breaks, modified nucleotides conjugated to a fluorochrome. The negative control was also carried out omitting TdT from the reaction mixture, while the positive control was obtained by pretreating sperm cells (about 0.5 × 10^6^) with 1 mg/mL of deoxyribonuclease I, not containing RNAse, at 37 °C for 60 min before staining. After incubation the cells were analysed by flow cytometry.

### 2.10. Detection of Lipoperoxidation

To evaluate lipoperoxidation, we used the probe BODIPY (581/591) C11 (Invitrogen, Thermo Fisher Scientific, Eugene, OR, USA). It is incorporated into cell membranes and responds to the attack of free oxygen radicals changing its spectrum emission from red to green, thus, it provided an estimate of the degree of peroxidation. Spermatozoa (an aliquot of 2 × 10^6^ cells) were incubated with 5 mM of the probe for 30 min in a final volume of 1 mL. Cells were washed with PBS, and after this the flow cytometric analysis was conducted. 

### 2.11. Measurement of Mitochondrial Superoxide Levels

The probe used to evaluate mitochondrial superoxide levels was the MitoSOX red mitochondrial superoxide indicator (Invitrogen, Thermo Fisher Scientific, Eugene, OR, USA). It penetrates into the mitochondria, is oxidized only by superoxide anion (not from other free radicals) and becomes highly fluorescent. Spermatozoa (an aliquot of 1 × 10^6^ cells) were incubated with 5 mM of the probe for 30 min in a final volume of 1 mL. After incubation the cells were analysed by flow cytometric analysis.

### 2.12. Statistical Analysis

The results are expressed as mean ± SEM throughout the study. Data were analyzed by one-way analysis of variance followed by the Duncan Multiple Range Test or Student’s *t*-test, as appropriate. SPSS 21 software for Windows 10 was used for statistical evaluation (SPSS Inc., Chicago, IL, USA). The statistical significance was accepted when the *p*-value was lower than 0.05.

## 3. Results

### 3.1. Sperm Parameters

The main sperm parameters of the 50 men enrolled in this study are shown in Table 1. All men had normal sperm parameters according to the WHO 2010 criteria. 

### 3.2. Effects of PFOA on Sperm Motility

**Figure 1:**
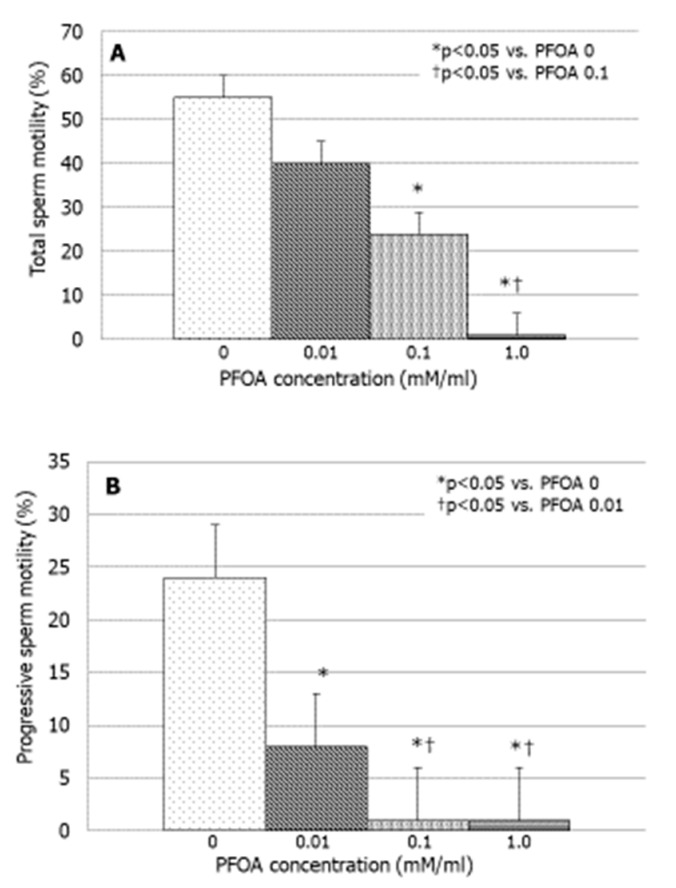
Effect of increasing concentrations of perfluorooctanoic acid (PFOA) on total (**A**) and progressive (**B**) sperm motility in vitro.

PFOA significantly inhibited total sperm motility at the concentration of 0.1 mM (*p* < 0.05 vs. PFOA 0) and suppressed to a greater extent this parameter with the concentration of 1 mM (*p* < 0.05 vs. PFOA 0.1 nM) (Figure 1A). The effects of PFOA were stronger on progressive sperm motility that was suppressed in a concentration-dependent manner. Indeed, the effect of PFOA on this parameter became significant at the concentration of 0.01 mM (*p* < 0.05 vs. PFOA 0), and it was significantly more pronounced at the concentrations of 0.1 and 1 mM (Figure 1B).

### 3.3. Effects of PFOA on Bio-Functional Sperm Parameters

**Figure 2:**
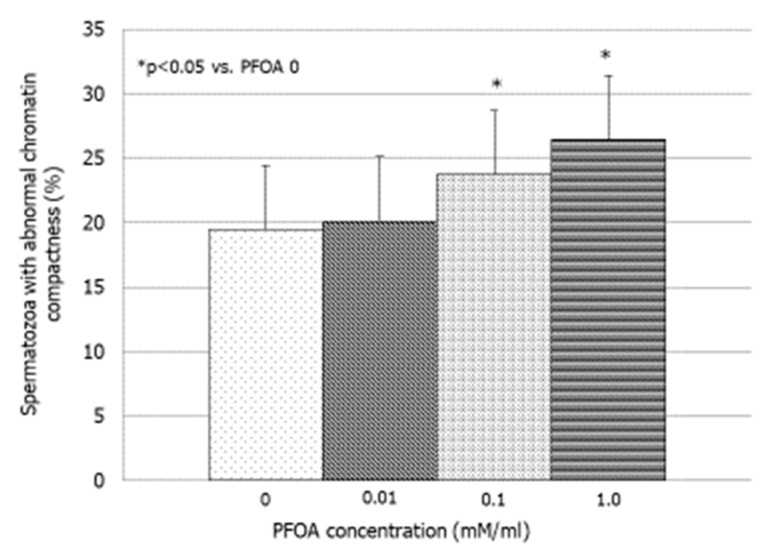
Effect of increasing concentrations of perfluorooctanoic acid (PFOA) on the degree of chromatin compactness in vitro.

PFOA significantly increased the percentage of spermatozoa with abnormal chromatin compactness at concentrations of 0.1 and 1 mM (*p* < 0.05 vs. PFOA 0) (Figure 2). No effect was observed on sperm viability, MMP, and DNA fragmentation (Table 2).

**Figure 3:**
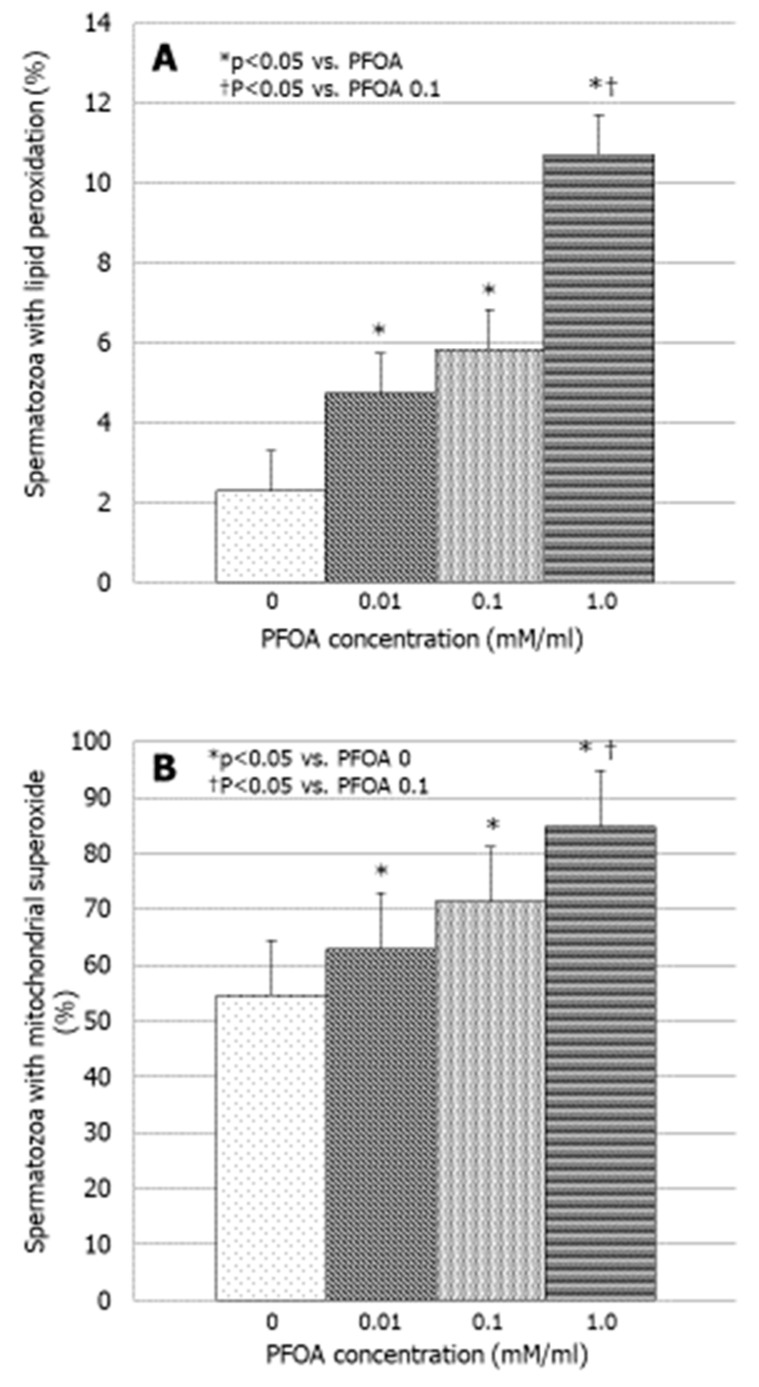
Effect of increasing concentrations of perfluorooctanoic acid (PFOA) on sperm membrane lipoperoxidation (**A**) and sperm mitochondrial superoxide production in vitro (**B**).

PFOA increased sperm lipid peroxidation in a concentration-dependent manner at all concentrations tested (*p* < 0.05 vs. PFOA 0) (Figure 3A). Finally, PFOA increased significantly the production of mitochondrial superoxide (*p* < 0.05 vs. PFOA 0). The effect became significant at the concentration of 0.01 mM and persisted as a such up to the concentration of 1 mM (*p* < 0.05 vs. PFOA 0) (Figure 3B). 

## 4. Discussion

Exposure to environmental and occupational chemicals may adversely affect male fertility. PFAS are ubiquitous and accumulate in the environment and the human body because of their stability and their ability to persist [29]. The effects of PFAS on male reproductive function are poorly understood because only a few studies have investigated the relationship between exposure to PFAS and male infertility with contradictory conclusions [14,30].

No correlation was reported between PFAS exposure and seminal fluid volume, sperm concentration, and total sperm count [31]. In contrast, other studies have shown that elevated PFOA levels negatively correlate with some conventional sperm parameters. Joensen and colleagues found an association between PFAS/PFOA exposure and sperm morphology in 105 young Danish men [30]. A study conducted on 212 men from the Veneto region (Italy) exposed to PFAS reported a significantly decreased progressive sperm motility [18]. An in vitro study demonstrated a significant increase in the percentage of immobile spermatozoa and a decrease in progressive sperm motility following incubation with PFOA [27]. However, other studies have not confirmed these results [31,32]. Raymer and colleagues found that PFOA levels in the blood and seminal fluid do not correlate with seminal fluid volume, sperm concentration, or motility [14]. Finally, a study conducted on men exposed to PFAS because of residence and lifestyle did not find alteration of sperm parameters [21].

The present study was undertaken to evaluate the effects of increasing concentrations of PFOA on human spermatozoa in vitro. After three hours of incubation, we found a profound negative effect of this compound on sperm motility and in particular on progressive motility, which was suppressed in a concentration-dependent manner until spermatozoa were completely immobilized. We previously reported the effects of other toxic substances on sperm motility, such as tumor necrosis factor-α, cigarette smoke extract, and benzo-a-pyrene, all of which were able to immobilize spermatozoa in vitro [33,34]. PFOA has proven to be equally effective. Similarly, high in vivo levels of PFOA in seminal fluid showed a significant negative correlation with sperm motility [18]. The exact mechanism(s), however, by which PFOA decreases sperm motility is unclear. PFOA can impair mitochondrial function, as observed for bisphenol-A, another endocrine disruptor that disrupts steroidogenesis, by altering the expression of key enzymes and transport proteins, such as StAR proteins [35]. The deleterious effects of PFOA recognize increased oxidative stress in the mitochondria that can alter sperm cytoskeleton and hinder ATP generation. All these events are known to reduce sperm motility [36].

We found that PFOA increased the percentage of spermatozoa with an altered degree of chromatin compactness in vitro but we did not find any significant effect on DNA fragmentation as shown in Table 2. Similarly, previous studies have shown that PFOA does not increase the percentage of spermatozoa with fragmented DNA [16,37]. The altered compactness of chromatin is not always sufficient to lead to DNA fragmentation. However, we cannot rule out that an increase in the rate of sperm DNA fragmentation can be achieved when spermatozoa are exposed to higher concentrations of PFOA [38] or when other PFAS are simultaneously present [26].

Sperm DNA damage can result from a concentration-dependent FAS-induced ROS hyperproduction [39]. Indeed, PFAS are known to exert their deleterious effects by increasing ROS production [40]. In particular, PFOA lowers considerably the total antioxidant capacity [39] and significantly increases the levels of 8-hydroxydeoxygunosine, a DNA catabolite after exposure to oxidative stress, in liver cells [41]. According to Hu and Hu, PFOA can overbalance superoxide dismutase, catalase, and glutathione reductase activities, and decreases the efficacy of glutathione-S-transferase and glutathione. The imbalance of the antioxidant system homeostasis due to ROS hyper-production can alter mitochondria function [42]. 

Furthermore, we found that PFOA causes peroxidation of sperm lipid membranes. This is in line with Yang and colleagues who showed that PFOA damages cell membrane by peroxiding membrane fatty acids [43]. Sperm membrane structure plays a key role in successful fertilization for its fluidity, flexibility, and functional activity [44], and plasma membrane lipid composition is directly related to sperm motility [45]. Unfortunately, membrane phospholipids are particularly susceptible to oxidative damage that can change membrane fluidity [46]. The amphipathic nature of PFAS suggests that their mechanism of action could focus precisely on the alteration of the membrane fluidity [47]. In particular, it was hypothesized that PFOA could increase membrane fluidity through an accumulation mechanism in the sperm cell membrane [27]. 

Finally, the results of the present study have shown that PFOA increased the production of mitochondrial superoxide. The oxidative balance of mitochondria is crucial for semen quality. Superoxide is a free radical generated by complexes I and III and high amounts of superoxide damage mitochondrial function. Similarly to what has been described by other authors such as Suh and colleagues on pancreatic β-cells, we found that the increased superoxide anion and total lipid peroxidation could also result in lipoperoxidation at the mitochondrial level [48], ensuing a potential mitochondrial that consequently affects motility, without altering MMP. Moreover, Sabovich and colleagues suggested that PFOA can act like other chemicals, such as zinc, that decrease mitochondrial respiratory activity but do not have any effect on MMP [27]. Therefore, PFOA could act on mitochondrial function also through the production of oxidative stress.

In conclusion, PFOA alters sperm motility up to immobilizing spermatozoa. Furthermore, it increased the oxidative stress, triggering lipid peroxidation also at the mitochondrial level. Therefore, the negative role of PFOA on sperm motility could be explainable through two different mechanisms. First, lipid peroxidation could also involve mitochondrial membranes, thus altering mitochondrial function without affecting MMP. Second, PFOA could alter cell membrane fluidity and, therefore, motility. According to these data, PFOA acts negatively on sperm function, and hence on male fertility. Moreover, the long-term effects of PFAS accumulated within the body are not predictable, thus it is necessary not to underestimate the effects of these toxic substances. 

## Figures and Tables

**Table 1:** Sperm parameters (mean ± SEM) of the 50 healthy normozoospermic men enrolled in this study.

Sperm Parameter	Values
Concentration (million/mL)	74 ± 8.5
Total count (million/ejaculate)	260 ± 30.8
Progressive motility (%)	30.6 ± 1.16
Total motility (%)	60 ± 6.5
Normal forms (%)	6.1 ± 0.47
Leukocytes (million/mL)	0.7 ± 0.04

**Table 2:** Effects of increasing concentrations of perfluorooctanoic acid (PFOA) on bio-functional sperm parameters.

PFOA Concentration (mM/mL)	0	0.01	0.1	1.0
Spermatozoa alive (%)	44.0 ± 3.3	34.9 ± 3.6	28.2 ± 8.4	27.1 ± 4.7
Spermatozoa in early apoptosis (%)	2.4 ± 1.6	2.7 ± 4.4	1.3 ± 3.5	4.8 ± 3.4
Spermatozoa in late apoptosis (%)	26.2 ± 6.9	32.4 ± 10.0	22.3 ± 3.7	29.9 ± 9.0
Spermatozoa with low mitochondrial membrane potential (%)	10.6 ± 1.7	14.6 ± 4.4	11.6± 4.1	20.0 ± 7.2
Spermatozoa with DNA fragmentation (%)	8.5 ± 3.4	9.1 ± 5.4	9.9 ± 1.2	10.7 ± 6.3

Results are expressed as mean ± SEM.

## Data Availability

Data are contained within the article.
